# Effects of distiller’s dried grains with solubles on enteric methane emissions in dairy and beef cattle: a meta-analysis

**DOI:** 10.3389/fvets.2024.1480682

**Published:** 2024-12-02

**Authors:** Muhammad Irfan Malik, Jianping Li, Maria Teresa Capucchio, Talal Hassan, Xuezhao Sun

**Affiliations:** ^1^The Innovation Centre of Ruminant Precision Nutrition and Smart Farming, Jilin Agricultural Science and Technology University, Jilin, China; ^2^Department of Veterinary Sciences, University of Turin, Turin, Italy; ^3^Jilin Inter-Regional Cooperation Centre for the Scientific and Technological Innovation of Ruminant Precision Nutrition and Smart and Ecological Farming, Jilin, China; ^4^Grasslands Research Centre, AgResearch Limited, Palmerston North, New Zealand

**Keywords:** distillers dried grains with solubles, methane, dairy cows, cattle, meta-analysis

## Abstract

**Introduction:**

Distiller’s dried grains with solubles (DDGS), a by-product of grain fermentation for ethanol production, are extensively used in livestock feed. Given their nutrient composition, DDGS could potentially influence methane (CH_4_) emissions, a significant greenhouse gas concern in ruminant production systems. This study utilized a multilevel random-effects meta-analysis to assess the impact of DDGS inclusion in cattle diets on CH_4_ production and yield.

**Methods:**

The literature search was conducted on 23 July 2024. Studies reporting CH_4_ emissions and dry matter intake (DMI) in cattle fed DDGS-based diets were identified, and data extraction was performed. The meta-analysis calculated the mean difference (MD) for DMI and CH_4_ yield and the relative mean difference (RMD) for CH_4_ production across the selected studies.

**Results:**

A total of *k* = 25 effect sizes from 10 studies were included in the DMI meta-analysis. DDGS had no significant effect on DMI in dairy or beef cattle (*p* = 0.770, MD = 0.070, 95% confidence interval [CI] from −0.420 to 0.561). For CH_4_ production, *k* = 24 effect sizes from 10 studies were analyzed, revealing no significant effect (*p* = 0.759, RMD = −1.045, 95% CI: from −8.025 to 5.935). Similarly, the meta-regression model indicated that the diet’s ether extract (EE) had no significant influence (*p* = 0.815, 95% CI from −1.121 to 1.409) on CH_4_ production. For CH_4_ yield, *k* = 23 effect sizes from 10 studies were included, with results showing no significant effect (*p* = 0.475, MD = −0.434 g/kg DMI, 95% CI: from −1.673 to 0.805). The regression model for the EE content of the diet also showed no significant impact on CH_4_ yield (*p* = 0.311, 95% CI: from −0.366 to 0.122).

**Discussion:**

The findings suggest that the inclusion of DDGS does not significantly affect DMI, enteric CH_4_ production, or CH_4_ yield in cattle. Moreover, the EE content in DDGS-containing diets does not significantly influence CH_4_ outcomes. These results indicate that DDGS can be incorporated into cattle diets without exacerbating CH_4_ emissions, contributing to sustainable livestock feeding practices.

## 1 Introduction

Distiller’s dried grains with solubles (DDGS) are widely utilized as a feed ingredient in livestock systems due to their abundant availability and robust nutritional profile. As a by-product of ethanol production through grain fermentation, DDGS is produced when two-thirds of the corn starch is converted to ethanol, leaving behind nutrients concentrated in the stillage (1). These nutrients are then recovered and processed into DDGS, resulting in a product with significantly enhanced nutritional content compared to the original grain. Specifically, the fermentation process triples the concentrations of protein, fiber, fat, and phosphorus in DDGS relative to corn, with typical DDGS compositions including 10–30% crude protein (CP), 4–12% fat, 12–36% neutral detergent fiber (NDF), and 0.3–0.9% phosphorus on a dry matter (DM) basis (2). The growing demand for bioethanol has led to increased production of DDGS, making it an increasingly important component of livestock feed. For instance, in 2023 alone, the United States exported 10.8 million metric tons of DDGS (3). The widespread adoption of DDGS in feed not only reduces reliance on imported soybean meal and cereals but also contributes to lowering the carbon footprint and enhancing food security (4). Corn DDGS is particularly well-established in dairy cattle diets, with inclusion levels of up to 300 g/kg of diet DM reported without adverse effects on milk yield (5). Due to its high protein content, DDGS is primarily used as a protein source for ruminants (6). However, there is limited research exploring the impact of DDGS on enteric methane (CH_4_) emissions in dairy and beef cattle.

Methane emissions are a critical issue in livestock production due to their significant contribution to greenhouse gases and their impact on climate change (7). Studies have shown mixed effects of DDGS inclusion on CH₄ emissions. In dairy cows, for instance, DDGS has been shown to reduce enteric CH₄ emissions without negatively impacting feed intake or milk production (8). However, DDGS inclusion has also been associated with increased manure CH_4_ emissions by up to 15% (9). In beef cattle, high levels of DDGS supplementation (40% on a DM basis) can reduce CH_4_ emissions but may simultaneously increase nitrous oxide emissions, highlighting a trade-off between different greenhouse gases (10).

Several studies have reported reductions in CH_4_ emissions when feeding DDGS to beef (11, 12) and dairy cattle (8). Hünerberg et al. (10) also reviewed that DDGS consistently resulted in lower CH_4_ emissions. The potential mechanism behind this reduction could be attributed to the higher fat content in DDGS (2), which can negatively affect ruminal fiber degradation, alter the acetate-to-propionate ratio, and reduce protozoa numbers, thereby decreasing CH_4_ production (8).

Due to inconsistencies in the literature, with some studies indicating that CH_4_ emissions are unaffected by varying levels of DDGS inclusion (13), animal nutritionists, policymakers, and farmers struggled to make informed decisions regarding the inclusion of DDGS as a CH_4_-mitigating feed ingredient in dairy and beef ration. Therefore, this meta-analysis was conducted to quantify the effect of DDGS inclusion in the diet on CH_4_ production and yield. Additionally, this study aimed to evaluate whether any reductions observed in CH_4_ emissions in dairy or beef cattle-fed DDGS are associated with the fat content of the diet.

## 2 Materials and methods

### 2.1 Search strategy and data processing

**Figure 1:**
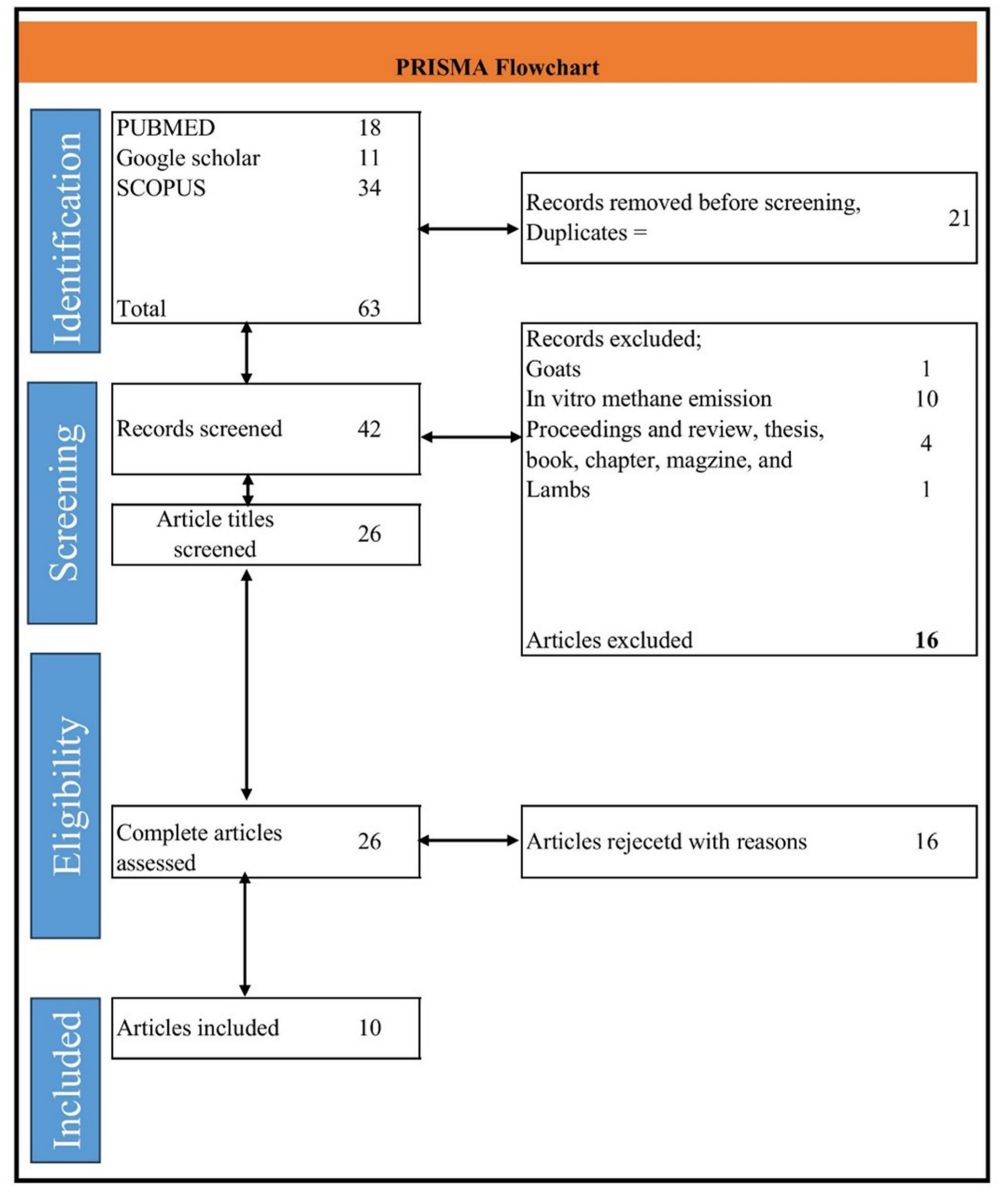
The preferred reporting items for systematic reviews and meta-analyses (PRISMA) flowchart for search strategy and details of study inclusion and exclusion.

The literature search was conducted on 23 July 2024, with no time restrictions applied. We selected two databases, PubMed^1^ and Scopus,^2^ along with Google Scholar, for our search. For PubMed and Scopus, we used the following keywords: DDGS OR dried distiller’s grains with solubles AND methane OR CH_4_ AND cattle OR cows OR beef OR steer OR cow OR heifer. For Google Scholar, the keywords were dried distiller’s grains with solubles OR DDGS AND methane. The detailed information on the search strategy is presented in the PRISMA flowchart (Figure 1) (14).

Only English-language, peer-reviewed articles were included, and studies reporting enteric CH_4_ emissions were selected. Articles that reported CH_4_ emissions from *in vitro* studies were excluded. Eligible studies had to involve dairy cattle, heifers, or beef cattle (either steers or heifers) and provide CH_4_ emission data. Data for CH_4_ emissions (g/day) were considered as CH_4_ production, and CH_4_ yield was reported as grams per kilogram of dry matter intake (DMI). We extracted data for CH_4_ production, CH_4_ yield, and DMI, acetate, propionate, and butyrate, along with sample size, standard deviation (SD), or standard error of the mean (SEM). For studies providing variance as SED, we used the RevMan calculator (Version 5.4, 15) to compute the SEM. Study characteristics, such as experimental design, diet composition (including % of forage in the diet, % of concentrate in the diet, NDF, EE, CP, starch, % of DDGS in the diet, and types of DDGS: wheat or corn), and types of animals were extracted (Table 1). For the study by Bernier et al. (16), where EE of the diet was not reported, it was calculated using the nutritional dynamic system (NDS) Professional Software. Methane production and yield reported in liters were converted to g/day and g/kg DMI, respectively. Liters per day were converted to grams per day, assuming that a mole of CH_4_, weighing 16.0 g, has a volume of 22.4 L (17).

**Table 1:** Database characteristics of primary studies included in the meta-analysis.

Reference	Methane quantification method	DDGS %	Animal	DOE	DDGS	Forage %	Concentrate %	EE %	CP %	NDF %	Starch %
McGinn et al. (11)	SF_6_	35.00	Beef	RCBD	Corn	60.00	40.00	5.10	17.40	42.50	
Bernier et al. (16)	SF_6_	10.70	Beef	RCBD	Corn, wheat	87.50	12.50	2.48	8.70	63.40	
Bernier et al. (16)	SF_6_	21.50	Beef	RCBD	Corn, wheat	76.50	23.50	3.04	11.40	58.60	
Benchaar et al. (8)	Respiratory chamber	10.00	Dairy cows	LSD	Corn	60.10	39.90	4.98	16.40	33.80	15.80
Benchaar et al. (8)	Respiratory chamber	20.00	Dairy cows	LSD	Corn	60.10	39.90	6.06	16.60	36.30	13.70
Benchaar et al. (8)	Respiratory chamber	30.00	Dairy cows	LSD	Corn	60.10	39.90	7.16	16.80	37.80	11.20
Hales et al. (32)	Respiratory chamber	15.00	Beef	LSD	Corn	10.00	90.00	4.80	14.30	16.80	58.70
Hales et al. (32)	Respiratory chamber	30.00	Beef	LSD	Corn	10.00	90.00	7.40	18.30	18.50	42.80
Hales et al. (32)	Respiratory chamber	45.00	Beef	LSD	Corn	10.00	90.00	8.30	20.20	18.70	39.10
Hales et al. (33)	Respiratory chamber	30.00	Beef	LSD	Corn	10.00	90.00	6.83	17.36	16.39	39.58
Hünerberg et al. (12)	Respiratory chamber	30.00	Beef	LSD	Corn	55.00	45.00	5.40	18.60	38.50	17.90
Hünerberg et al. (12)	Respiratory chamber	30.00	Beef	LSD	Wheat	55.00	45.00	3.70	23.50	33.90	16.80
Hünerberg et al. (46)	Respiratory chamber	40.00	Beef	LSD	Corn	8.00	92.00	5.40	19.60	27.90	34.70
Hünerberg et al. (46)	Respiratory chamber	40.00	Beef	LSD	Wheat	8.00	92.00	3.10	23.10	24.50	31.90
Castillo-Lopez et al. (47)	Indirect calorimetry	20.00	Dairy cows	LSD	Corn	50.70	49.30	3.90	17.10	38.10	21.40
Castillo-Lopez et al. (47)	Indirect calorimetry	20.00	Dairy cows	LSD	Corn	50.70	49.30	3.30	17.10	37.90	21.30
Castillo-Lopez et al. (47)	Indirect calorimetry	20.00	Dairy cows	LSD	Corn	50.70	49.30	3.60	17.10	38.00	21.30
Judy et al. (48)	Indirect calorimetry	20.00	Dairy cows	LSD	Corn	58.97	41.03	3.38	17.20	34.70	23.20
Judy et al. (48)	Indirect calorimetry	20.00	Dairy cows	LSD	Corn	58.97	41.03	4.76	16.90	35.10	21.90
Garnsworthy et al. (13)	Infrared analyzer	9.55	Dairy cows	LSD	Wheat	61.85	38.15	4.16	18.85	35.20	19.22
Garnsworthy et al. (13)	Infrared analyzer	19.15	Dairy cows	LSD	Wheat	62.25	37.75	4.15	18.97	37.65	17.70
Garnsworthy et al. (13)	Infrared analyzer	29.00	Dairy cows	LSD	Wheat	62.70	37.30	4.20	19.07	40.20	16.15
Garnsworthy et al. (13)	Infrared analyzer	6.80	Dairy cows	LSD	Wheat	61.10	38.90	4.19	19.00	34.80	27.20
Garnsworthy et al. (13)	Infrared analyzer	22.00	Dairy cows	LSD	Wheat	61.80	38.20	4.91	19.00	35.40	21.80
Garnsworthy et al. (13)	Infrared analyzer	27.10	Dairy cows	LSD	Wheat	62.00	38.00	5.51	19.00	35.70	20.00

DDGS, distiller’s dried grains with solubles; DOE, design of experiment; EE, ether extract; NDF, neutral detergent fiber; CP, crude protein of the diet; LSD, Latin square design; RCBD, randomized control block design; SF_6_, sulfur hexafluoride.

### 2.2 Data analysis

The analysis utilized mean difference (MD) as the outcome measure for DMI and CH_4_ yield (treatment mean – control mean). Methane production was calculated as relative mean difference (RMD) = [(treatment mean – control mean)/(control mean)] × 100. The RMD, a dimensionless variable, was used to account for large variations and is particularly useful for expressing percentage changes in methane production, which is of greater interest to readers (18). The standardized mean difference (SMD) for volatile fatty acids is a statistical technique commonly employed in meta-analyses to compare and synthesize findings from different studies that use varying measurement scales (19, 20). To calculate the SMD, the mean of the control group is subtracted from the mean of the treatment group, and the result is divided by the pooled standard deviation (19). A positive SMD indicates that the treatment group had a higher mean than the control group, while a negative SMD suggests the opposite. We applied a multilevel random-effects model to address the dependency of effect sizes from the same study. This three-level meta-analytical model is appropriate for handling dependence and heterogeneity among studies. In this model, effect sizes extracted from the same study are considered nested within higher levels, making it suitable for scenarios with varying degrees of variation both within and between studies. The multilevel meta-analysis technique provides more precise effect sizes of treatment effects and helps identify sources of heterogeneity. The variance distribution in the model is as follows: level 1 = sampling variance, level 2 = effect sizes extracted from the same study, and level 3 = variance between studies. By accounting for the varying levels of variation within and between studies, the multilevel meta-analysis technique can provide more precise effect sizes of treatment effects and aid in identifying the sources of heterogeneity (21, 22). We applied an equal effect model for acetate, as the limited number of studies prevented the multilevel model from converging. Convergence refers to the optimizer’s ability to identify the best-fitting parameters for the applied model. Successful convergence occurs when the algorithm effectively minimizes or maximizes the target function. Conversely, failure to converge can result from issues, such as poorly specified models, insufficient data, or constraints, that hinder the optimizer’s ability to find an optimal solution. Additionally, a subgroup analysis was conducted based on the types of DDGS fed to the animals, with subgroups created for wheat and corn DDGS.

Heterogeneity (*τ*^2^) was estimated using the DerSimonian-Laird estimator (23), and the *I*^2^ statistic (24) was reported and calculated as follows:


$$I^{2}=\frac{Q-\left(k-1\right)}{Q}\times 100$$

where Q is the *χ*^2^ statistic and *k* is the number of studies included in the meta-analysis.

A prediction interval for the true outcomes was also provided (25). The Knapp and Hartung adjustment method was used for the tests and confidence intervals (26). Potential outliers and influential studies were assessed using studentized residuals and Cook’s distances (27). Meta-regression was performed to test the hypothesis that CH_4_ emissions decreased with increased EE contents in the diet, with EE included as a continuous variable in the multilevel random-effects meta-regression model. Studies with studentized residuals larger than the 100 × [1–0.05/(2 × *k*)] percentile of a standard normal distribution were considered potential outliers (Bonferroni correction with two-sided *α* = 0.05 for *k* studies). Studies with Cook’s distances larger than the median plus 6 times the interquartile range were deemed influential. Sensitivity analyses assessed the robustness of the results by removing statistical outliers with 95% confidence intervals lying outside the pooled effect size (27). Funnel plot asymmetry was checked using the rank correlation test (28) and the regression test by Sterne and Egger (29), with the standard error of observed outcomes as the predictor. Data analysis was performed using R (version 4.4.0) (30) and the metafor package (version 4.6.0) (31).

## 3 Results

### 3.1 Database characteristics

The data analysis included 6 studies on beef cattle and 4 studies on dairy cattle, yielding 11 effect sizes for beef and 14 effect sizes for dairy. The experimental design was a randomized complete block design (RCBD) in two studies and a Latin square design (LSD) in the remaining eight. Two types of DDGS were used: wheat-based DDGS in three studies and corn-based DDGS in seven. Methane quantification methods varied, with the sulfur hexafluoride (SF_6_) trace gas technique used in one study, an infrared analyzer in another, indirect calorimetry in two, and a respiratory chamber in five (Table 1).

On average, the inclusion rate of DDGS was 29.74% for beef cattle and 19.54% for dairy cattle, with concentrate levels at 64.54 and 41.28%, respectively (Table 2). For dairy cattle, forage averaged 58.71% of the diet, with CP at 17.79%, NDF at 36.47%, and starch at 19.41%. In contrast, beef cattle diets had a higher DDGS content (29.74%) and more variable forage levels (35.45%), with CP averaging at 17.49%, NDF lower at 32.69%, and starch higher at 35.18% (Table 2).

**Table 2:** Descriptive statistics for the dietary characteristics of the studies included in the meta-analysis.

Variables	Mean	Minimum	Maximum	SD	Missing
Dairy cattle					
DDGS	19.54	6.80	30.0	6.887	0
Forage	58.71	50.70	62.70	4.496	0
Concentrate	41.28	37.30	49.30	4.496	0
EE	4.590	3.300	7.160	1.080	0
CP	17.79	16.40	19.07	1.090	0
NDF	36.47	33.80	40.20	1.806	0
Starch	19.41	11.20	27.20	4.161	0
Beef cattle					
DDGS	29.74	10.70	45.00	10.57	0
Forage	35.45	8.000	87.50	31.39	0
Concentrate	64.54	12.50	92.00	31.39	0
EE	5.050	2.480	8.300	1.893	0
CP	17.49	8.700	23.50	4.537	0
NDF	32.69	16.58	63.40	16.58	0
Starch	35.18	16.80	58.70	13.59	3

All units are in %, otherwise mentioned. SD, standard deviation; DDGS, distiller’s dried grains with solubles; EE, ether extract; NDF, neutral detergent fiber; CP, crude protein.

A summary of the multilevel random-effects meta-analysis and meta-regression for DMI and methane production and yield is provided in Table 3, offering a concise overview of the statistical results.

**Table 3:** Summary statistics of the multilevel random effects meta-analysis and meta-regression for dry matter intake and methane production and yield.

Variables	Effect size	SE	*T*-value	DF	*p-*value	95% CI	Q	*I^2^*	Egger’s test *p*-value
DMI	0.070	0.238	0.295	24	0.770	−0.420 to 0.561	39.56	39.34	0.001
Types of DDGS									
Corn	0.146	0.229	0.638	14	0.529	−0.328 to 0.612	–	–	–
Wheat	−0.509	0.328	−1.548	7	0.135	−1.191 to 0.172	–	–	–
Corn:Wheat	1.327	0.979	1.355	1	0.189	−0.704 to 3.358	–	–	–
CH_4_ Production	−1.045	3.374	−0.309	23	0.759	−8.025 to 5.935	21.5	0	0.469
Types of DDGS									
Corn	−3.502	5.059	−0.692	13	0.496	−14.02 to 7.019	–	–	–
Wheat	−0.243	7.179	−0.034	7	0.973	−15.17 to 14.68	–	–	–
Corn:Wheat	4.347	12.26	0.354	1	0.726	−21.14 to 29.84	–	–	–
Ether extract	0.144	0.611	0.611	23	0.815	−1.121 to 1.409	–	–	–
CH_4_ Yield	0.434	0.597	−0.726	22	0.475	−1.673 to 0.805	48.00	54.16	0.161
Types of DDGS									
Corn	−0.835	0.836	−0.998	12	0.330	−2.580 to 0.910	–	–	–
Wheat	0.903	1.308	0.690	7	0.498	−1.826 to 3.632	–	–	–
Corn:Wheat	−0.359	2.399	−0.149	1	0.882	−5.364 to 4.646	–	–	–
Ether extract	−0.122	0.117	−1.036	22	0.311	−0.366 to 0.122	–	–	–

SE, standard error; DF, degree of freedom (number of effect size); 95% CI, 95% confidence interval; DDGS, distiller’s dried grains with solubles; DMI, dry matter intake.

### 3.2 Dry matter intake

**Figure 2:**
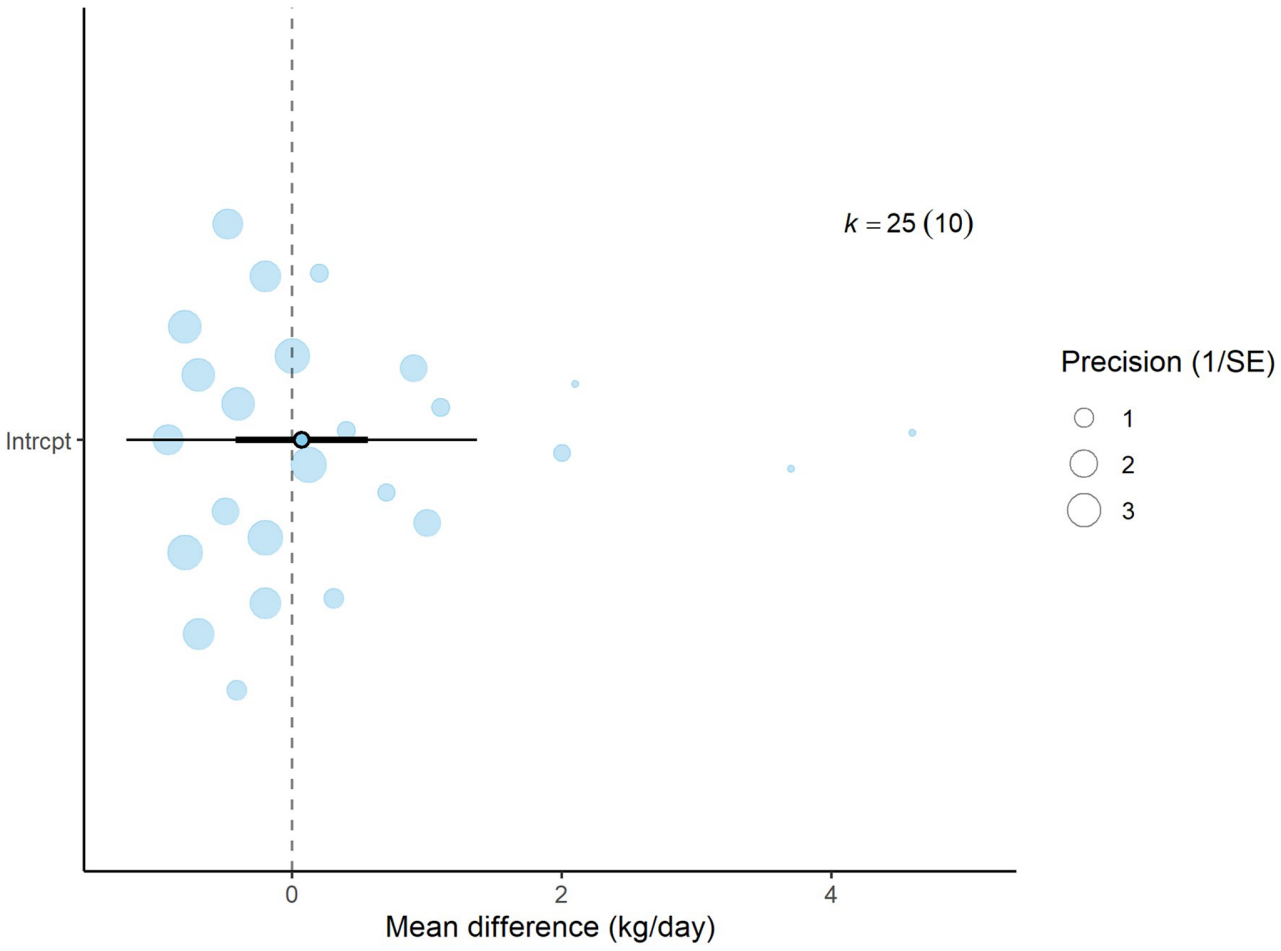
Orchard plot for dry matter intake (DMI): The overall effect size from a multilevel random-effects meta-analysis of 25 effect sizes is centered on zero, with a 95% confidence interval (CI) spanning the line of no effect (dotted line). The thick black horizontal line indicates the prediction interval, while the dotted vertical line marks the line of no effect.

**Figure 3:**
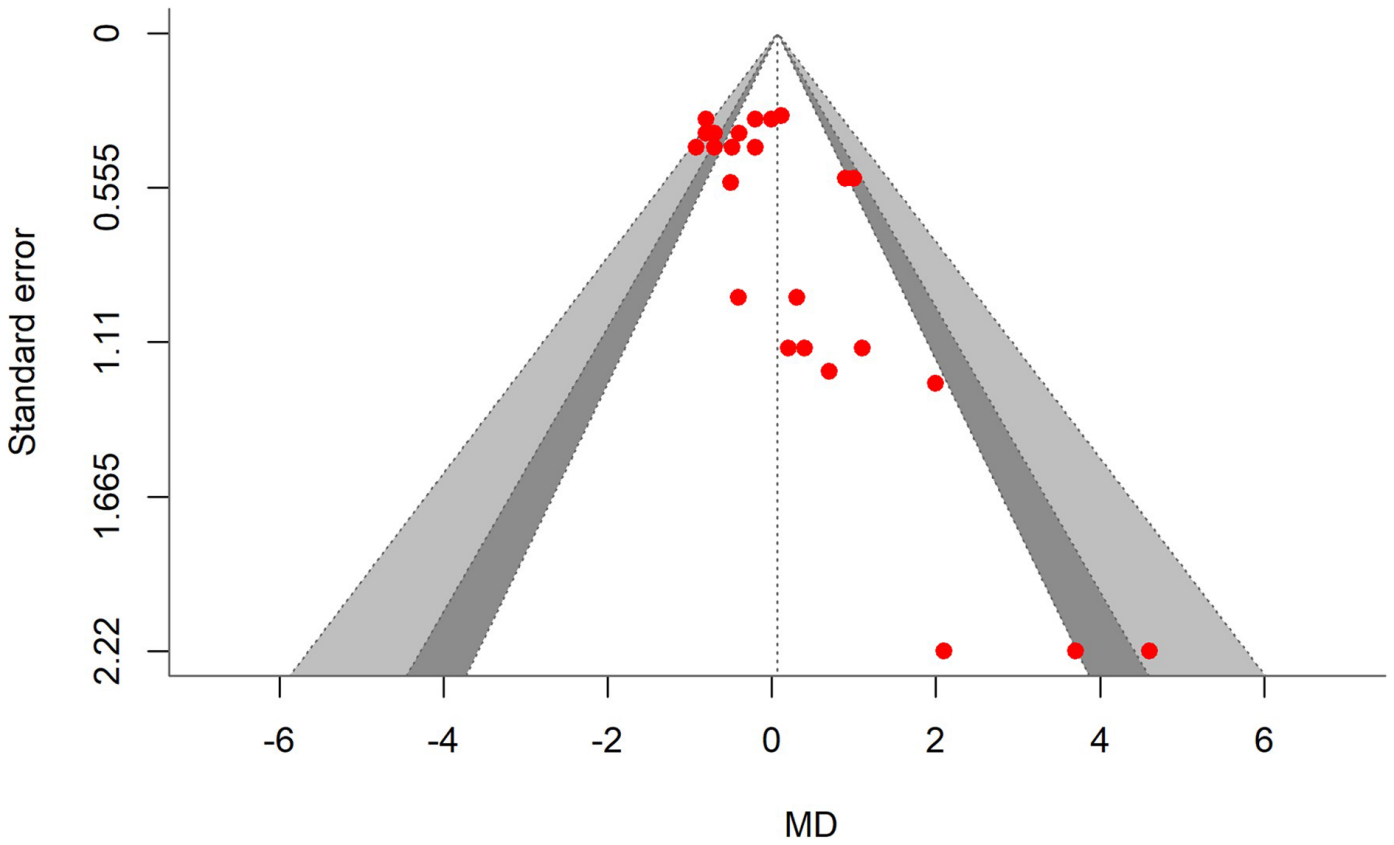
Contour-enhanced funnel plot showing asymmetrical distribution of effect sizes around the standard error, indicating bias in the dry matter intake (DMI) meta-analysis. MD, the mean difference.

A total of *k* = 25 effect sizes from 10 studies were included in the analysis. The observed mean differences ranged from −0.92 to 4.60, with 48% of the effect sizes being negative. The multilevel random-effects meta-analysis indicated that DDGS had no significant effect on DMI in dairy or beef cattle (*p* = 0.770, MD = 0.070, 95% CI: from −0.420 to 0.561). An orchard plot illustrating the observed outcomes and the effect size from the multilevel random-effects model is presented in Figure 2. The subgroup analysis for the different types of DDGS was also non-significant (*p* > 0.05) for corn, wheat, or a mixture of both. The effect sizes were as follows: corn DDGS (*p* = 0.529, MD = 0.146, 95% CI = from −0.328 to 0.612), wheat DDGS (*p* = 0.135, MD = −0.509, 95% CI = from −1.191 to 0.172), and a mixture of corn and wheat DDGS (*p* = 0.189, MD = 1.327, 95% CI = from −0.704 to 3.358). The Q-test revealed heterogeneity among the true outcomes (Q = 39.56, *p* = 0.023, *τ*^2^ = 0.148, *I*^2^ = 39.34%). Since the heterogeneity (*I*^2^) was below 40% and the primary outcome was non-significant, meta-regression was not conducted, as adding covariates would be meaningless. An examination of studentized residuals showed no outliers, with no values exceeding ±3.09. Additionally, Cook’s distances indicated that none of the studies were overly influential. The funnel plot of the effect sizes, shown in Figure 3, indicated potential asymmetry, supported by the rank correlation and Egger’s regression tests (*p* = 0.009 and *p* = 0.001, respectively).

### 3.3 Methane production

**Figure 4:**
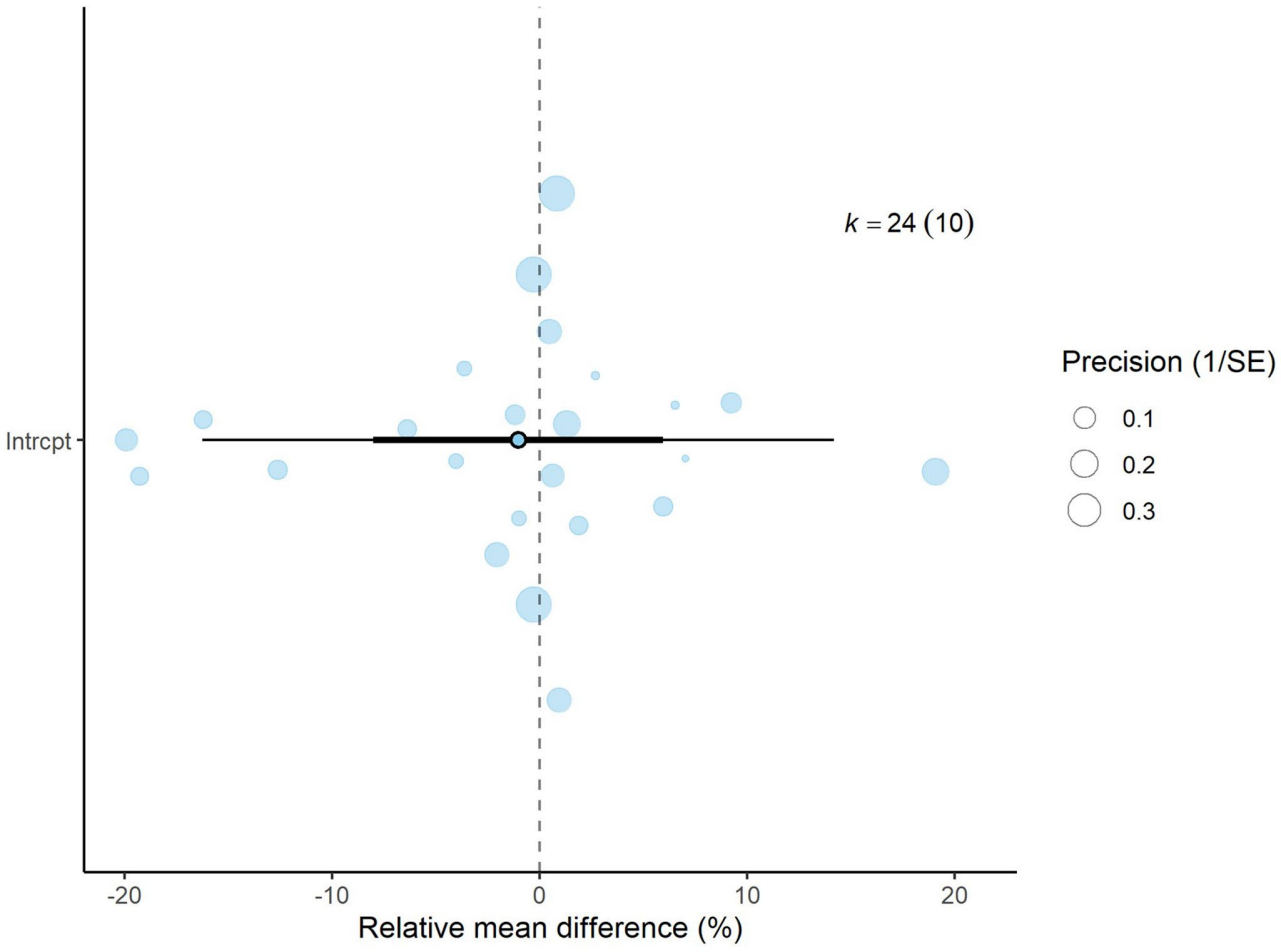
Orchard plot for methane production % (relative mean difference): The overall effect size from a multilevel random-effects meta-analysis of 24 effect sizes is centered on zero, with a 95% confidence interval (CI) spanning the line of no effect (dotted line). The thick black horizontal line represents the prediction interval, while the dotted vertical line marks the line of no effect.

**Figure 5:**
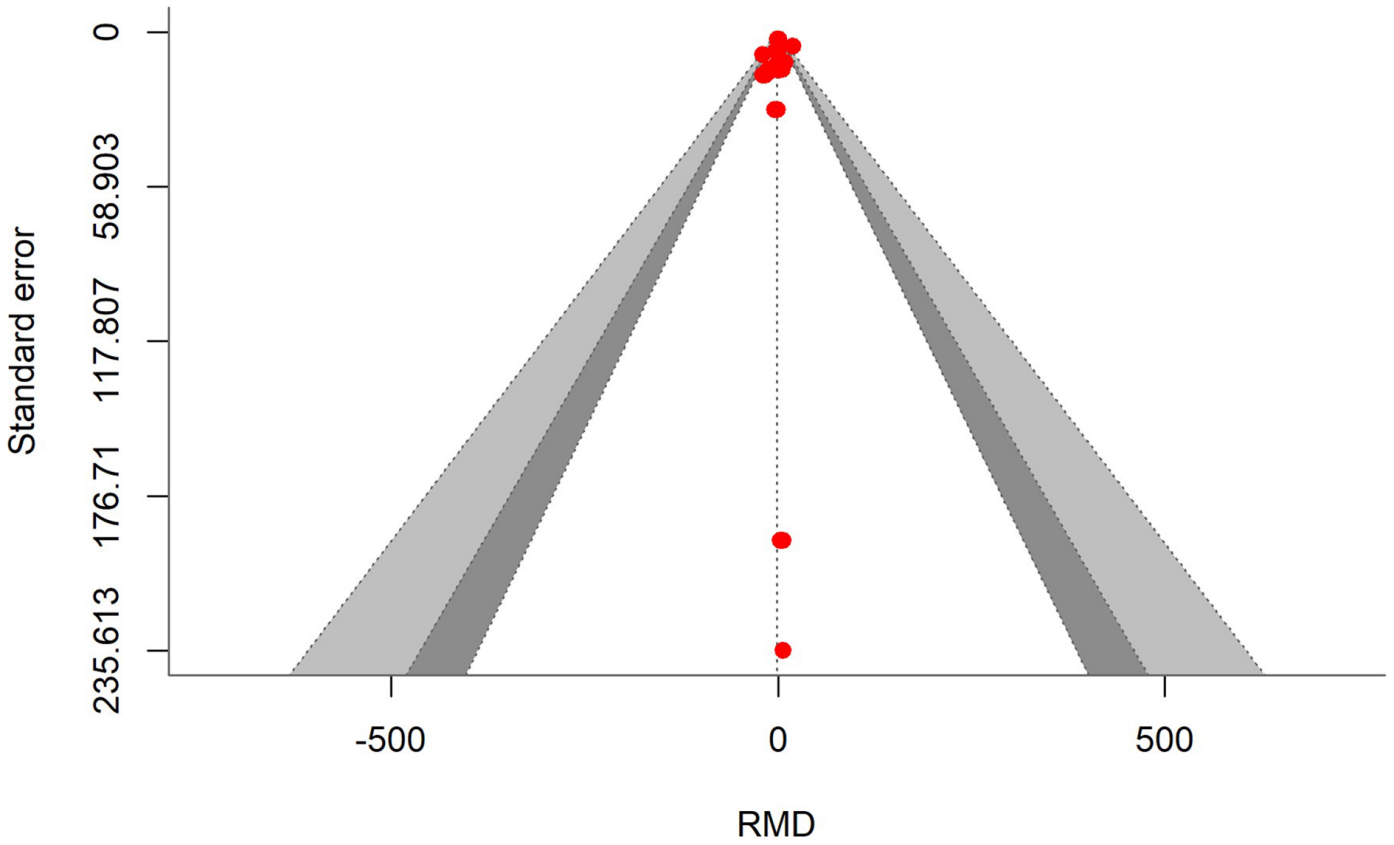
Contour-enhanced funnel plot showing symmetrical distribution of effect sizes around the standard error, indicating no bias in the methane production meta-analysis.

A total of *k* = 24 effect sizes from 10 studies were analyzed. Sensitivity analysis identified a treatment with 45% distiller grains as an outlier and overly influential, leading to its exclusion from the final analysis (32). The observed RMD was −1.045%, with 52% of the effect sizes being positive. Methane production was found to be non-significant (*p* = 0.759, RMD = −1.045, 95% CI: from −8.025 to 5.935). An orchard plot showing the observed outcomes and the prediction interval is presented in Figure 4. The subgroup analysis of different types of DDGS showed no significant impact on methane production. For corn-based DDGS, the effect size was non-significant (*p* = 0.496, RMD = −3.502, 95% CI = from −14.02 to 7.019). Similarly, wheat-based DDGS had no notable effect (*p* = 0.937, RMD = −0.243, 95% CI = from −15.17 to 14.68). The combination of corn and wheat DDGS also showed no significant influence (*p* = 0.726, RMD = 4.347, 95% CI = from −21.14 to 29.84). The regression model indicated that the EE of the diet had no significant effect on CH_4_ production (*p* = 0.815, 95% CI: from −1.121 to 1.409), with an increase of 0.144% in CH_4_ production per unit increase in EE. The Q-test suggested homogeneity among the true outcomes (Q = 21.5, *p* = 0.550, *τ*^2^ = 0, *I*^2^ = 0), indicating no heterogeneity. The funnel plot in Figure 5 showed no asymmetry, as confirmed by the rank correlation and Egger’s regression tests (*p* = 0.549 and *p* = 0.469, respectively).

### 3.4 Methane yield

**Figure 6:**
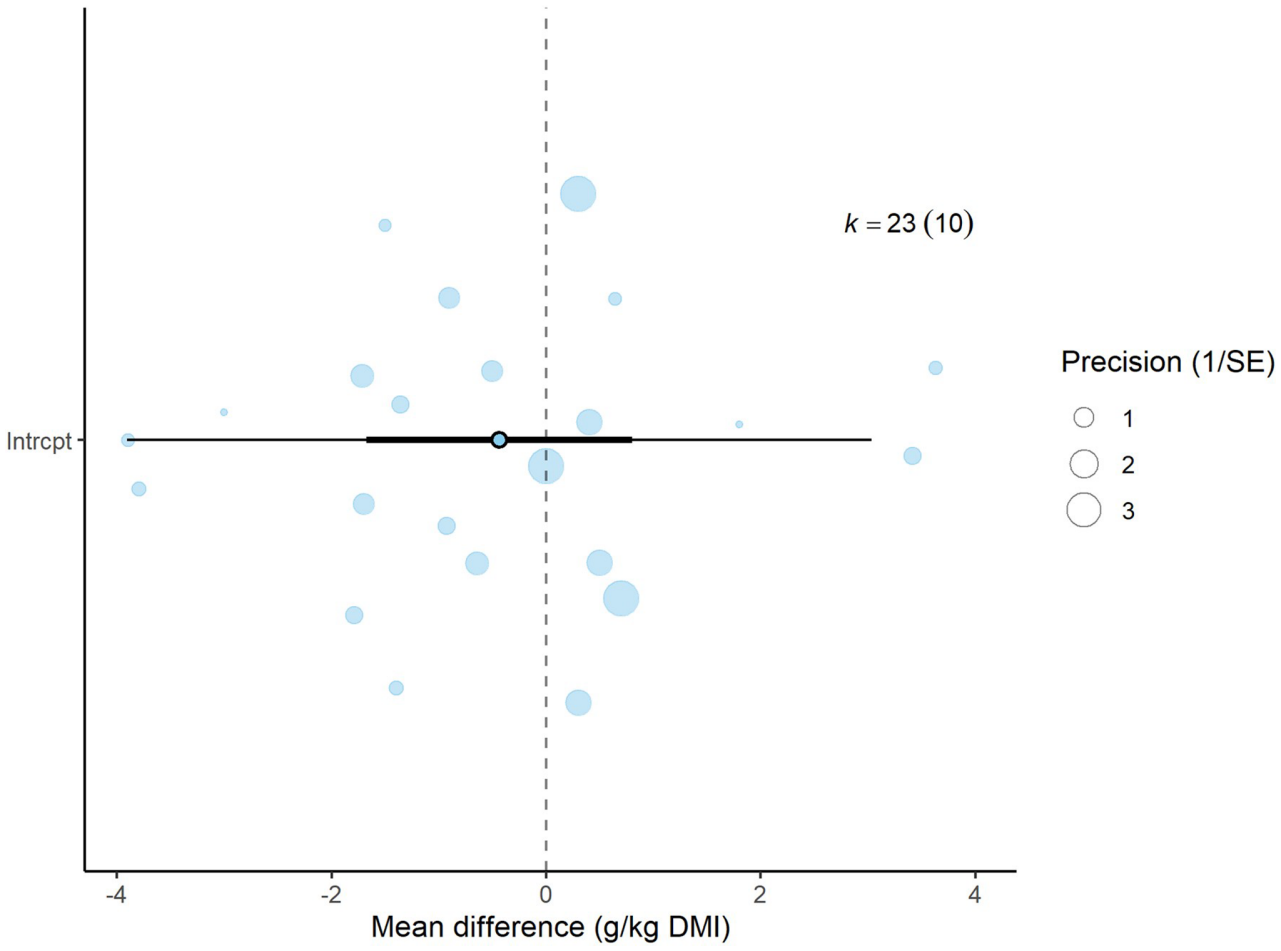
Orchard plot for methane yield: The overall effect size from a multilevel random-effects meta-analysis of 23 effect sizes is centered on zero, with a 95% confidence interval (CI) spanning the line of no effect (dotted line). The thick black horizontal line represents the prediction interval, while the dotted vertical line marks the line of no effect. SE, standard error.

**Figure 7:**
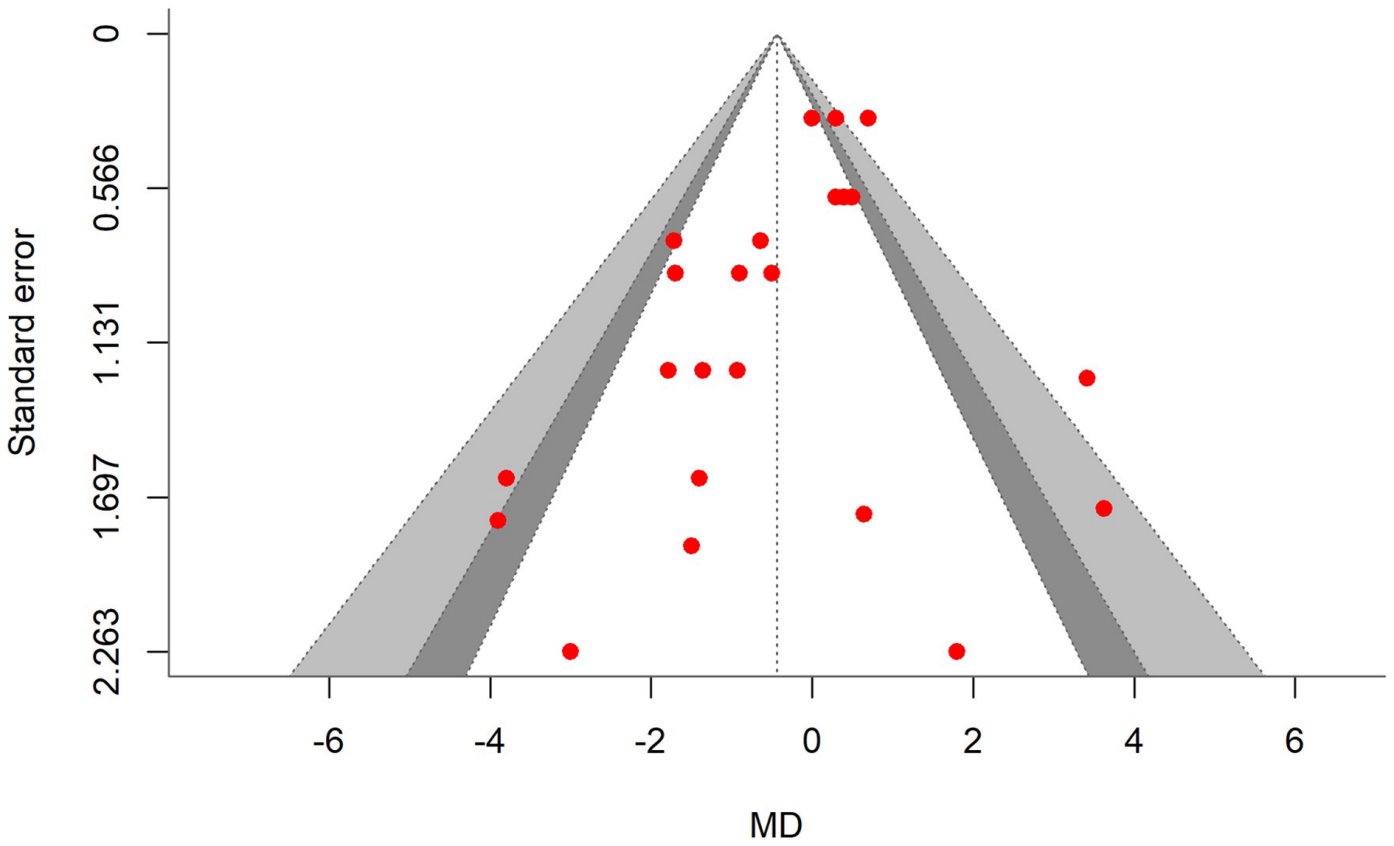
Contour-enhanced funnel plot for studies included in the methane yield meta-analysis: symmetrical distribution of effect sizes around the standard error indicates no bias.

**Figure 8:**
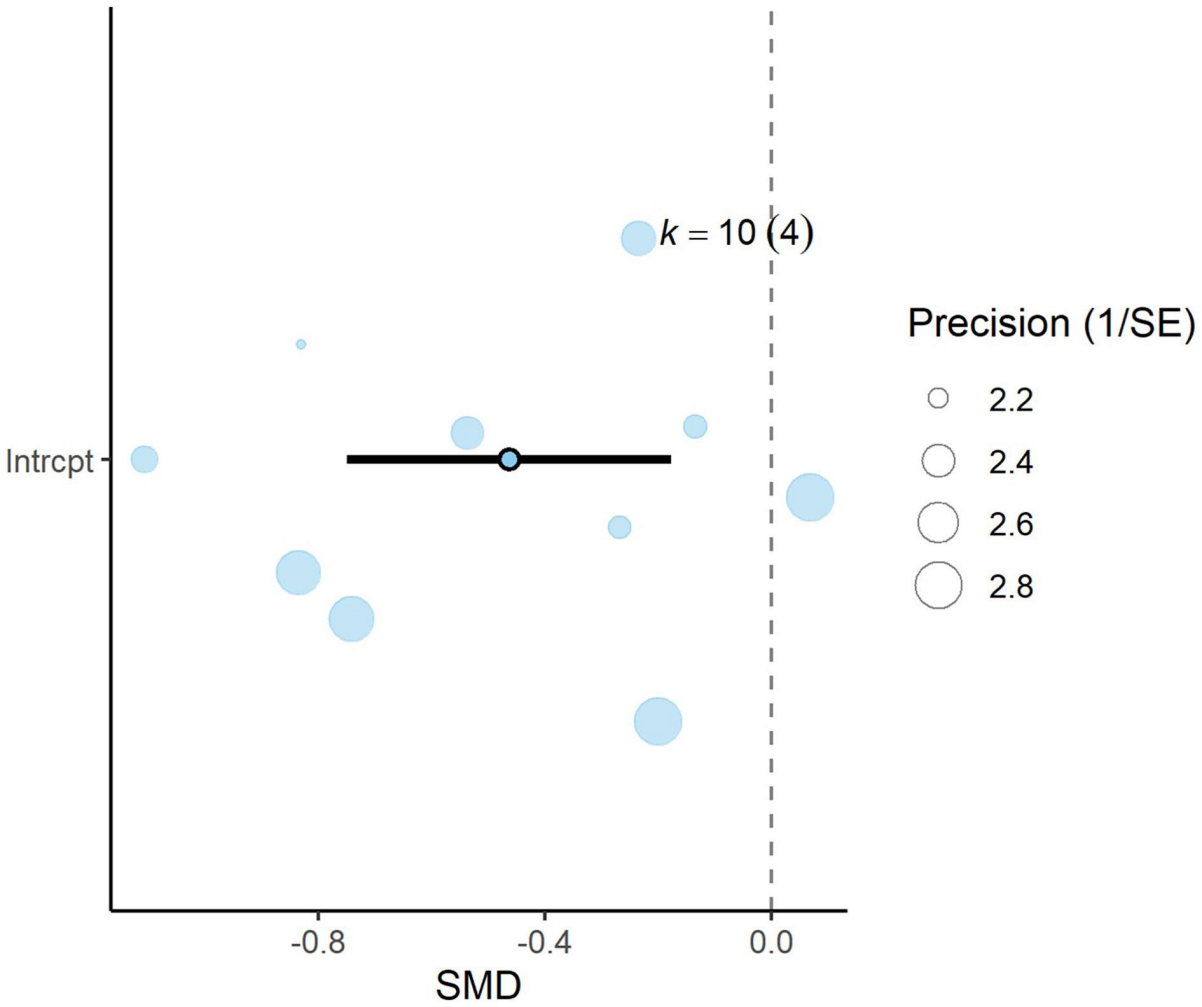
Orchard plot for acetate: The overall effect size from a multilevel random-effects meta-analysis of 10 effect sizes is centered on zero, with a 95% confidence interval (CI) spanning the line of no effect (dotted line). The thick black horizontal line represents the prediction interval, while the dotted vertical line marks the line of no effect. SE, standard error.

A total of *k* = 23 effect sizes from 10 studies were included in the analysis. Sensitivity analysis identified two treatments with 30 and 45% distiller grains as outliers, which were subsequently removed from the final analysis (32). The observed mean differences for CH_4_ yield ranged from −3.90 to 3.63, with 57% of the effect sizes being negative. Methane yield was found to be non-significant (*p* = 0.475, MD = −0.434 g/kg DMI, 95% CI: from −1.673 to 0.805). An orchard plot depicting the observed outcomes and the prediction interval is shown in Figure 6. The regression model for EE indicated no significant effect on CH_4_ yield (*p* = 0.311, 95% CI: from −0.366 to 0.122), with a − 0.122 g/kg DMI increase in CH_4_ yield per unit increase in EE. The subgroup analysis for types of DDGS suggests that DDGS types have no significant effect on methane yield. The effect sizes were as follows: corn DDGS (*p* = 0.330, MD = −0.835, 95% CI = from −2.580 to 0.910), wheat DDGS (*p* = 0.498, MD = 0.903, 95% CI = from −1.826 to 3.632), and a mixture of corn and wheat DDGS (*p* = 0.882, MD = −0.359, 95% CI = from −5.364 to 4.646). The Q-test indicated heterogeneity among the true outcomes (Q = 48, *p* = 0.001, *τ*^2^ = 0.55, *I*^2^ = 54.16%). The funnel plot in Figure 7 showed no significant asymmetry, supported by the rank correlation and Egger’s regression tests (*p* = 0.183 and *p* = 0.161, respectively) (Figure 8).

### 3.5 Acetate

A total of *k* = 10 effect sizes from four studies were included in the analysis. The observed SMD for acetate was found to be significant (*p* = 0.005, SMD = −0.463, 95% CI: from −0.749 to −0.176). Subgroup analysis by DDGS type indicated that corn DDGS significantly decreased rumen acetate production, with an effect size of (*p* = 0.001, SMD = −1.048, 95% CI: from−1.526 to −0.570) (Table 4). In contrast, wheat DDGS showed no significant difference (*p* = 0.176, SMD = −0.313, 95% CI: from −0.801 to 0.173). Due to the substantially reduced acetate production, a meta-regression was conducted to identify potential moderators influencing acetate levels. We found that increasing the inclusion level of DDGS in dairy cattle diets significantly reduced acetate (*p* = 0.005, SMD = −0.024, 95% CI: from −0.040 to −0.009). Similarly, the inclusion of EE had a significant effect on rumen acetate production (*p* = 0.002, SMD = −0.102, 95% CI: −0.159 to 0.046). The Q-test indicated no significant heterogeneity among the true outcomes (Q = 8.28, *p* = 0.506, *τ*^2^ = 0, *I*^2^ = 0%). Funnel plot asymmetry was also non-significant, as supported by both the rank correlation and Egger’s regression tests (*p* = 0.216 and *p* = 0.461, respectively).

**Table 4:** Summary statistics for the equal effect meta-analysis and meta-regression for rumen acetate production.

Variables	Effect size	SE	*T*-value	DF	*p*-value	95% CI	Q	*I^2^*	Egger’s test *p-*value
Acetate	−0.463	0.126	−3.654	9	0.005	−0.749 to −0.176	8.28	0.0	0.461
Ether extract	−0.102	0.025	−4.099	9	0.002	−0.159 to 0.046	–	–	–
DDGS %	−0.024	0.006	−0.360	9	0.005	−0.040 to −0.009	–	–	–
Types of DDGS									
Corn	−1.048	0.207	−5.054	4	0.001	−1.526 to −0.570	–	–	–
Wheat	−0.313	0.211	−1.483	4	0.176	−0.801 to 0.173	–	–	–

SE, standard error; DF, degree of freedom (number of effect size); 95% CI, 95% confidence interval; DDGS, distiller’s dried grains with solubles. All effect sizes are expressed as standardized mean difference (SMD).

### 3.6 Butyrate

**Figure 9:**
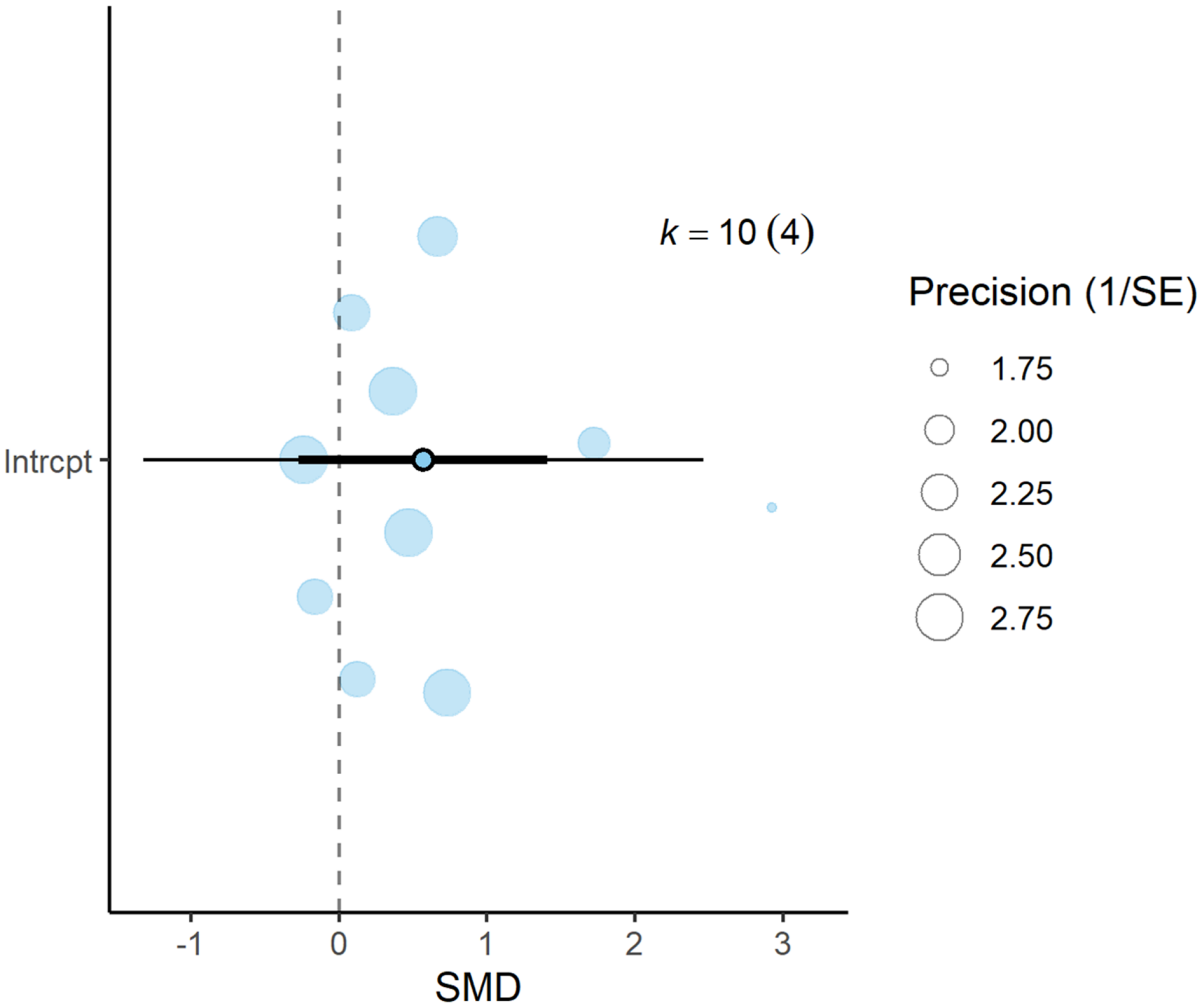
Orchard plot for butyrate: The overall effect size from a multilevel random-effects meta-analysis of 10 effect sizes is centered on zero, with a 95% confidence interval (CI) spanning the line of no effect (dotted line). The thick black horizontal line represents the prediction interval, while the dotted vertical line marks the line of no effect. SE, standard error.

A total of *k* = 10 effect sizes from four studies were analyzed. Rumen butyrate production was found to be non-significant (*p* = 0.159, SMD = 0.569, 95% CI: from −0.270 to 1.409) (Figure 9). Subgroup analysis by DDGS type showed no significant impact on butyrate production (Table 5). For corn-based DDGS, the effect size was non-significant (*p* = 0.102, SMD = 0.784, 95% CI: from −0.198 to 1.766), and wheat-based DDGS also showed no notable effect (*p* = 0.384, SMD = 0.389, 95% CI: from −0.586 to 1.365). The regression model indicated that the EE of the diet had no significant effect on CH₄ production (*p* = 0.067, SMD = 0.131, 95% CI: from −0.011 to 0.274). The Q-test suggested significant heterogeneity among the true outcomes (Q = 32.33, *p* = 0.0002, τ^2^ = 0.563, *I*^2^ = 76.58%). The funnel plot showed asymmetry; the rank correlation test was non-significant (*p* = 0.216), while Egger’s regression test was significant (*p* = 0.006).

**Table 5:** Summary statistics for the multilevel random effects meta-analysis and meta-regression for rumen butyrate production.

Variables	Effect size	SE	*T*-value	DF	*p-*value	95% CI	Q	*I^2^*	Egger’s test *p-*value
Butyrate	0.569	0.371	1.532	9	0.159	−0.270 to 1.409	32.33	76.58	0.006
Ether extract	0.131	0.063	2.082	9	0.060	−0.011 to 0.274	–	–	–
DDGS %	0.022	0.012	1.729	9	0.117	−0.006 to 0.051	–	–	–
Types of DDGS									
Corn	0.784	0.426	1.840	4	0.102	−0.198 to 1.766	–	–	–
Wheat	0.389	0.432	0.919	4	0.384	−0.586 to 1.365	–	–	–

SE, standard error; DF, degree of freedom (number of effect size); 95% CI, 95% confidence interval; DDGS, distiller’s dried grains with solubles. All effect sizes are expressed as standardized mean difference (SMD).

### 3.7 Propionate

**Figure 10:**
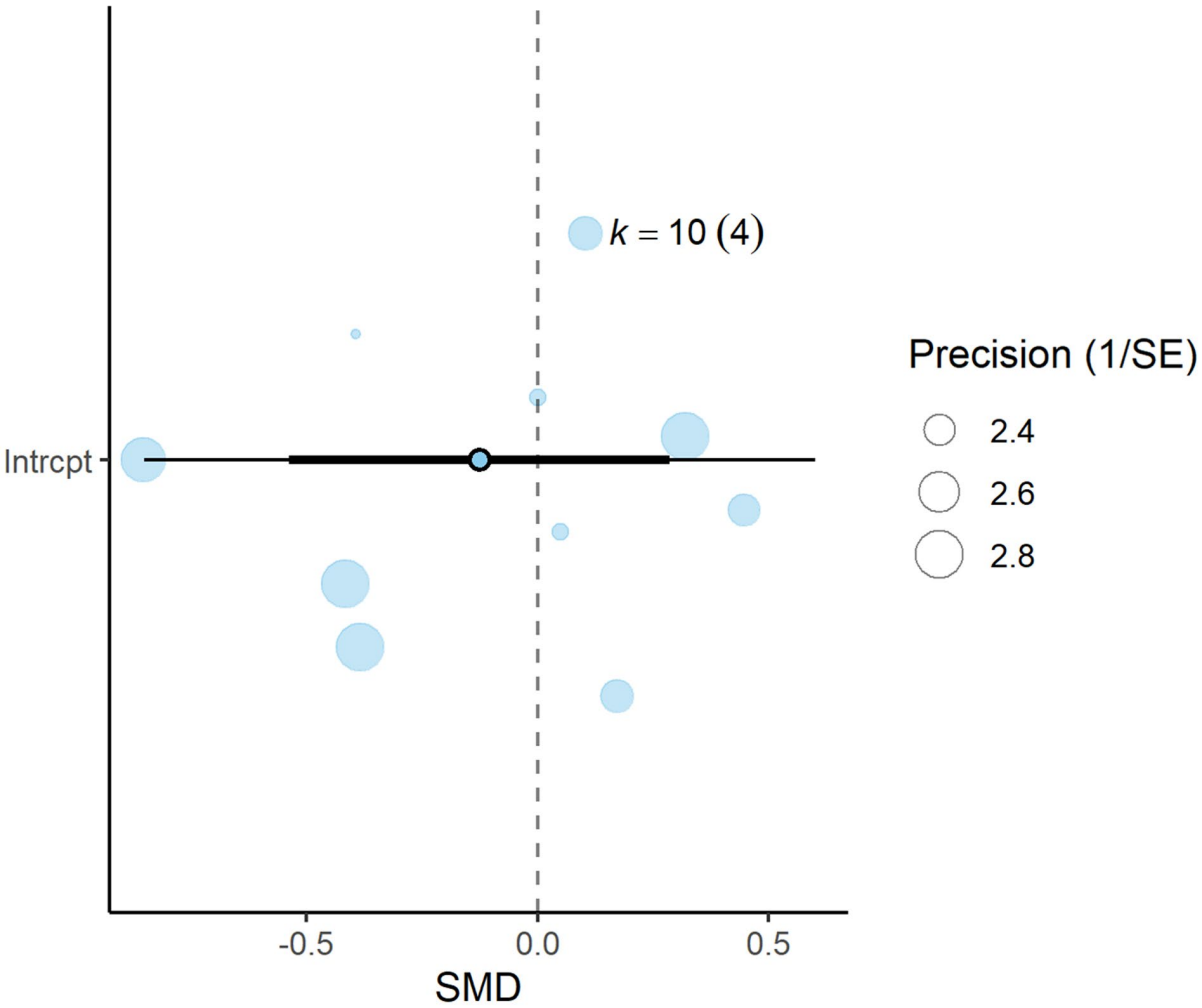
Orchard plot for propionate: The overall effect size from a multilevel random-effects meta-analysis of 10 effect sizes is centered on zero, with a 95% confidence interval (CI) spanning the line of no effect (dotted line). The thick black horizontal line represents the prediction interval, while the dotted vertical line marks the line of no effect. SE, standard error.

A total of *k* = 10 effect sizes from four studies were included in the analysis. The observed SMD for acetate was found to be non-significant (*p* = 0.508, SMD = −0.125, 95% CI: from −0.538 to −0.286) (Figure 10). Subgroup analysis by DDGS type indicated that corn DDGS had no effect on rumen propionate production (*p* = 0.622, SMD = 0.116, 95% CI: from −0.408 to 0.641). Similarly, wheat DDGS showed no significant difference (*p* = 0.139, SMD = −0.382, 95% CI: from −0.920 to 0.155) (Table 6). EE also had no significant effect on rumen propionate production (*p* = 0.913, SMD = −0.004, 95% CI: from −0.087 to 0.079). The Q-test indicated no significant heterogeneity among the true outcomes (Q = 9.99, *p* = 0.350, τ^2^ = 0.029, *I*^2^ = 30.73%). Funnel plot asymmetry was non-significant, as confirmed by the rank correlation and Egger’s regression tests (*p* = 1.0 and *p* = 0.417, respectively).

**Table 6:** Summary statistics for the multilevel random effect meta-analysis and meta-regression for rumen propionate production.

Variables	Effect size	SE	*T*-value	DF	*p-*value	95% CI	Q	*I^2^*	Egger’s test *p-*value
Propionate	−0.125	0.182	−0.689	9	0.508	−0.538 to −0.286	9.99	15.5	0.417
Ether extract	−0.004	0.036	−0.111	9	0.913	−0.087 to 0.079	–	–	–
DDGS %	−0.006	0.005	−1.187	9	0.265	−0.018 to 0.005	–	–	–
Types of DDGS									
Corn	0.116	0.227	0.511	4	0.622	−0.408 to 0.641	–	–	–
Wheat	−0.382	0.233	−1.639	4	0.139	−0.920 to 0.155	–	–	–

SE, standard error; DF, degree of freedom (number of effect size); 95% CI, 95% confidence interval; DDGS, distiller’s dried grains with solubles. All effect sizes are expressed as standardized mean difference (SMD).

## 4 Discussion

Methane emissions from livestock production are a significant contributor to climate change, posing a major challenge among atmospheric pollutants. It is well established that CH_4_ emissions are influenced by both the quantity and type of nutrients fermented in the rumen. Typically, CH_4_ production increases with DMI, and the specific nutrients fermented play a crucial role in rumen methanogenesis.

Our findings suggest that DDGS have no significant effect on DMI in both dairy and beef cattle, consistent with previous studies in beef cattle (11, 33) and dairy cattle (16). However, this is in contrast to other studies that observed increased DMI when DDGS replaced soybean meal and corn in dairy cattle diets (34). The discrepancy may be attributed to our study’s focus on literature that specifically evaluates enteric CH_4_ emissions, potentially excluding studies that might have reported positive effects on DMI without examining CH_4_ outcomes (8). This limitation highlights the need for a more comprehensive analysis that includes a broader range of studies.

Our meta-analysis found that both CH_4_ yield and production were non-significant, indicating that DDGS does not influence CH_4_ emissions in dairy or beef cattle. This finding contrasts with some studies that suggest DDGS can impact these emissions (11, 33). For instance, research has shown that feeding DDGS to dairy cows can mitigate enteric CH_4_ emissions without negatively affecting intake and milk production (8). In beef cattle, DDGS inclusion has also been associated with reduced CH_4_ emissions (11, 33), attributed to the high EE (EE) content of DDGS (12.7% of DM), which can range from 2.0 to 5.1% of DM (11).

The reduction in CH_4_ production in these studies is often linked to increased EE supply from DDGS, which affects ruminal fiber degradation, the ratio of acetate to propionate, and protozoa numbers. These factors collectively contribute to lower CH_4_ production. However, our meta-regression analysis did not find a significant influence of EE on CH₄ production or yield, contradicting the hypothesis that higher fat content from DDGS would reduce CH_4_ emissions. This suggests that the relationship between dietary fat content in DDGS-supplemented cows and CH_4_ emissions may be more complex than previously thought and warrants further investigation. Another potential mechanism could be related to sulfur concentration. Buckner et al. (35) analyzed 1,200 DDGS (corn =400 and wheat = 800) samples from six ethanol processing facilities over 10 months, reporting an average sulfur content of 0.78%. Higher sulfur content may reduce CH_4_ emissions by redirecting ruminal H_2_ from methanogenesis for CH_4_ production (36) toward hydrogen sulfide (H_2_S) production. Hydrogen sulfide has been shown to inhibit methanogenic archaea directly (37). The activity of sulfate-reducing bacteria (SRB) depends on the availability of H_2_ and sulfate levels, as these bacteria use sulfate as a terminal electron acceptor in anaerobic respiration (38, 39). By increasing the sulfate level in the rumen, the capacity of SRB to outcompete methanogens as an H_2_ sink is enhanced, which could further reduce CH₄ emissions (40). This suggests that sulfur and sulfate levels in DDGS may influence the microbial dynamics, favoring pathways that reduce CH₄ production. Our findings suggest that dietary fat is not responsible for CH_4_ reduction in DDGS-supplemented cows. The reduction in CH_4_ emissions reported in some studies might be associated with higher sulfur contents, and variations in sulfur levels due to regional and processing differences could explain the differing results across studies.

The findings of the current meta-analysis suggest that acetate production decreases significantly in cows supplemented with DDGS, which aligns with previous studies in dairy cattle (8, 41, 42). This reduction in acetate may be linked to a decline in ruminal fiber digestion and a decrease in the ruminal degradability of hay as the proportion of DDGS in the diet increases (8). These results are further supported by the meta-regression model, which shows a linear decrease in acetate production with increasing DDGS inclusion. In contrast, butyrate and propionate production were not influenced by the percentage or type of DDGS. The literature shows inconsistencies regarding butyrate and propionate production. Leupp et al. (43) reported a decrease in acetate molar proportion alongside an increase in propionate, with no effect on butyrate in beef cattle. Meanwhile, Anderson et al. (44) observed a numerical decrease in acetate and increases in both propionate and butyrate molar proportions in dairy cows fed DDGS diets. There was evidence of publication bias in both DMI and butyrate. This bias may be linked to the unilaterally skewed effect sizes observed in the meta-analysis. Additionally, meta-analyses with a smaller number of studies are more susceptible to publication bias than those with a larger number of studies, which can affect the reliability and representativeness of the findings (45). The implications of our findings for livestock management and CH_4_ mitigation are significant. Although DDGS may not consistently reduce CH_4_ emissions, their diet inclusion offers other nutritional benefits, such as improved nitrogen utilization. However, it is essential to consider the environmental impact of increased nitrogen excretion when evaluating the overall sustainability of DDGS in cattle diets (8). Future research should focus on identifying the conditions under which DDGS can effectively reduce CH_4_ emissions and exploring the underlying mechanisms in greater detail. Studies should also investigate the potential relationship between CH_4_ reduction and sulfur content in cattle diets, particularly when supplemented with DDGS. Given that DDGS is rich in both fats and sulfur, it is important to distinguish the individual effects of these components on CH_4_ emissions and overall cow health. Understanding how sulfur and fats interact within the rumen and their combined impact on CH_4_ reduction will be crucial in developing more sustainable cattle diets that mitigate environmental impact while ensuring animal health. Additionally, addressing the significant variability observed in the literature could provide clearer insights into the role of DDGS in CH_4_ mitigation.

## 5 Conclusion

Our meta-analysis indicates that the inclusion of DDGS has no significant impact on DMI in dairy or beef cattle in studies that evaluated enteric CH_4_ emissions. Similarly, DDGS supplementation in cattle diets does not influence enteric CH_4_ production or yield. Furthermore, the EE content of diets containing DDGS does not significantly affect CH_4_ production or yield in these cattle.

These findings have important implications for livestock producers and policymakers seeking to balance the nutritional benefits of DDGS with the need for effective CH_4_ mitigation strategies. Continued research is essential to refine our understanding of DDGS’s role in CH_4_ emissions and to explore alternative dietary strategies that can contribute to more sustainable livestock production systems.

A key limitation of this meta-analysis is the relatively small number of studies available on enteric CH_4_ emissions and rumen volatile fatty acids in dairy and beef cattle supplemented with DDGS. This limited dataset may reduce the statistical power and generalizability of the results, as fewer studies can increase variability and the potential for bias. Future research involving a larger body of studies would be valuable in validating and expanding upon the conclusions drawn here.

## Data Availability

The data analyzed in this study is subject to the following licenses/restrictions: Inquiries regarding the original data for the meta-analysis can be directed to Muhammad Irfan Malik, dr.irfan279@gmail.com.

## Footnotes

^1^https://pubmed.ncbi.nlm.nih.gov

^2^https://www.scopus.com/home.uri
